# Graduate Study on the Continent—Austria.—IV

**Published:** 1908-12-05

**Authors:** 


### HOSPITAL ADMINISTRATION.

### CONSTRUCTION AND ECONOMICS.

### GRADUATE STUDY ON THE CONTINENT—AUSTRIA.—IV.

THE GRADUATE STUDENT AT VIENNA.

WE have shown in the preceding articles that graduate study at Vienna has many attractions and also some drawbacks. Of the latter, perhaps the greatest is the fact that there are few courses which are really practitioners' courses. We quite admit, as most Vienna students will contend, that very many of the courses, especially those for which the fees are relatively high, are attended only by graduates. That is quite true. But it is equally true that all these courses are open to the advanced but unqualified student as well. We do not say that this is a drawback in university courses. These cannot be other than mixed courses, for the university must, first of all, concern itself with its matriculates. But we do contend that for private courses, as at Berlin, for example, there appears to be little opportunity. Such private courses are given, but from what we have seen of them they cannot be held to compare with the graduate courses given at Hamburg.

There is, however, every prospect that this matter will be taken up vigorously by the teachers at Vienna and that full use will be made of the large amount of excellent clinical material available for study. The Vienna Aerztekammer, animated by the example of the Berlin colleagues, appointed a committee of eight members in 1906 to consider the question of regular post-graduate courses. To this committee the medical faculty of the University joined five representatives, and the joint committee carefully considered the matter from all aspects. The result of its deliberations was the organisation of a series of real post-graduate courses, the first of which were given in October 1906. These courses are open to city practitioners who are members of the Aerztekammer, but, as in Germany, foreign graduates may, by courtesy, be enrolled as members. The courses take place in October, and applications should be made early in the September preceding. The fee for each course varies from 3 to 6 kronen, payable in advance. Members of the Aerztekammer have the first choice of any course, and for the most part the number of members in a course is strictly limited. The admission of a foreign graduate who is not a member of the Aerztekammer is therefore only possible when there is a vacancy. Such graduates must pay a fee varying from 10 to 20 kronen for each course, not to the Aerztekammer, but to the lecturer or demonstrator himself, and their admission to a course is subject to his approval. The following courses are given, all of them very good: Dr. Bum, Massage and Physical Therapy; Prof. Chiari, Laryngology and Rhinology; Prof. Chobrak, Practical Obstetrics; Dr. Clairmont, On Fractures and Dislocations; Prof. Freiherr von Eiselsberg, Recent Advances in Surgery; Prof. Escherich, On Diphtheria; Prof. Finger, The Diagnosis and Treatment of Gonorrhœa; Prof. von Frankl-Hochwart, The Diagnosis of Nervous Diseases; Prof. von Frisch, Treatment of Diseases of the Urinary Tract; Dr. Von Jagié, Clinical Microscopy and Blood Examinations; Dr. Kleinböck, Radiology; Dr. Koschier, Practical Laryngology; Prof. Lorenz, Orthopædics; Prof. Paltauf, Serumtherapy; Prof. Politzer, Otology; Prof. Schauta, Obstetrics and Gynæcology; Dr. Schmidt, The Diseases of the Stomach; Prof. Schnabl, Practical Ophthalmology; Prof. Spiegler, Syphilology; Prof. Ritter von Jauregg, Psychiatry.

From this list it will be seen that the practitioner is well catered for. All the courses are modelled on the German system—that is to say, they are thoroughly practical, given by specialists, and in every way suited to the needs of the graduate student. We strongly recommend the graduate visitor to Vienna to join one of these classes and to judge for himself of the excellence of the teaching.

For the student who wishes to do free-lance work, a method of study that has many advantages, who does not desire to join a special course but to see as much of methods as possible, and who can, moreover, afford the time and the money for a lengthy stay at Vienna, this centre holds out many attractions. The concentration of clinical material allows him to see a vast deal in a day, but he will do well to confine his energies to one or two clinics. As it is impossible here to deal with all the Vienna hospitals, as our space does not allow of it, we confine ourselves to pointing out what are the attractions of the best-known institution—the large

ALLGEMEINES KRANKENHAUS AT VIENNA.

This is an old, rambling hospital, a little village in itself, interspersed with gardens and courtyards, and containing numerous clinics, at the head of which are well-known University professors. When it was founded in 1784 by the Emperor Joseph II. (whose statue adorns the first Hof) the quarter in which it stands was still a suburb. Now Alsersgrund is one of the city wards, and the hospital is much too small to cope with the number of patients from the city alone. It can accommodate 2,000 patients in a series of 100 wards, but the buildings are old and worn, and the internal arrangements do not favourably impress the visitor who is familiar with modern hospitals. Here the foreign graduate can work, either as a free-lance or as an assistant. Assistantships are not easy to get, for the competition is relatively keen; nevertheless a certain number are held yearly by foreign graduates, mostly American. From early morning to midday the student can occupy himself here in attending lectures, demonstrations, clinics, or operations. In the first medical clinic lecture-room Prof. Van Noorden gives his lecture on medicine four times weekly. In the three surgical clinics Professors Eiselsberg, Hochenegg, and Frank lecture and operate. Presentation of the visitor's card is quite sufficient to admit him to any lecture or operating-theatre. In the latter he is required to put on overalls. Lectures and theatres are usually crowded. A few days' attendance here will soon show the student what is worth cultivating and what he can strike off his list. If he is interested in surgical work he will probably haunt the first and second surgical clinics, and drop in occasionally to attend those excellent and enjoyable demonstrations which Professor Lorenz gives on orthopædic subjects. Professor Lorenz and his three chief assistants work in a small, stuffy out-patient department in the second Hof, which is usually overcrowded by patients and auditors. The professor, one of the most charming of personalities, lectures in English as well as in German, and he is by no means singular in doing so. Many of the professors and privat-docenten speak English fluently, and at no other centre can the student who is unacquainted with German get along so well. This, however—if we may be allowed to say so—is not altogether an advantage. With the best will and intention in the world, the English lecture of a German privat-docent is not equal to his German lectures—if we except such fluent English speakers as Professor Lorenz and Dr. Spitzy, of Graz, and some others whose English demonstrations are every whit as good as those which they give in their mother-tongue. We may again repeat that the graduate student who is not willing to learn enough German to follow a lecture or demonstration in that language had much better stay at home, where he will probably find far better teaching than he can obtain from English-speaking teachers at Vienna.

In all the clinics the student will find himself heartily welcomed. He is allowed to see as much as he pleases, his questions are courteously answered, and everyone is ready to help him. For practical study and for the man who can depend on himself—who observes, who learns instead of waiting to be taught—it is an excellent centre, and the Allgemeines Krankenhaus is an excellent institution. Its numerous clinics are an unfailing mine of novelties and interesting cases. Thus at the post-mortem room the student will daily find a mass of material. The demonstrations, which are open to any student to attend, are given daily in the morning and are always well worth attending, for one may see on one occasion more than one has been able to observe during a term's assiduous attendance at a London post-mortem room. Its value as a centre of instruction is unsurpassed by any other place in Vienna, just as, in our opinion, Prof. Lorenz's demonstrations are unsurpassed by any others given in the clinics of the general hospital.

There is a small reading-room attached to the Allgemeines Krankenhaus (entrance in the ophthalmological block; present card to the attendant). Usually a small fee is charged for making use of it. It does not contain much; thus the only English paper represented is the *Lancet*, many weeks old, though there are several American journals. The main German and Austrian medical papers are filed here. There is also a small library of standard professional works.

The gynæcological wards of the General Hospital have lately been moved into the fine new gynæcological block facing the side entrance to the hospital on the Spital and Lazarettestrasse (opposite the Café Klinik). This block contains the two departments, gynæcological and obstetrical, under the charge of Professor Roshorn and Schauta respectively. Although the wards are by no means perfect as specimens of hospital construction, they are yet very interesting and will well repay a visit. The operating-theatres are large and fine, and every facility is given to the visitor to see operations.

Beyond the Allgemeines Krankenhaus the student should make a point of visiting the newer institutions. Chief among these are the fine Serum Therapeutic Institute, the Billroth Hospital, the Rothschild Hospital, the Asylum, and the anatomical and physiological institutes. Every one of these will repay inspection, and at each he can see much that is of interest. For practical study a constant attendance at the Polyclinic Institute will prove the most useful, together with frequent visits to the clinics at the Allgemeines, whether surgical, medical, or special. Here, as at Berlin, a good time-table is a necessity. The student should provide himself with a copy of the official programme of lectures (20 hellers; any bookseller) which will give him every information regarding times of lectures, clinics to visit, etc., and will prove useful in other respects. Then he should map out his time-table, bearing in mind that although there is more concentration at Vienna there is also more overlapping than at other large centres. Thus he will have to miss one or two lectures or demonstrations daily, while if he wishes to devote himself to the post-mortem room or the polyclinic he will find it practically impossible to put in more than the early morning lectures.

It would be difficult to say for what special branch of study Vienna holds out extraordinary attractions. When one goes merely by amount of material and excellence of teaching one would put ophthalmology and laryngology first. In such subjects as pathology, serum therapy, otology, and diseases of children, it compares favourably with most other centres. For pure medicine, gynæcology, or surgery, the student will not find that it surpasses Berlin.

To sum up, the student who is acquainted with Berlin, Munich, and Leipsig will probably be very greatly disappointed with Vienna as a graduate centre. On the other hand, the graduate who has not been to these centres, and to whom the Austrian capital is the first foreign centre he has touched at, it will appear a veritable paradise so far as clinical material is concerned. It is all a matter of comparison, and in some respects also one of opinion, in which individual liking and preference will largely influence the verdict. For ourselves, while we have found the time spent there by no means wasted, and while we are fully conscious that it offers many attractions, we do not esteem it as highly as Berlin or Leipsig, and, in the matter of pure graduate courses, we do not think it can compare with Hamburg. Nevertheless, it is a centre which no graduate, who has the time and means at his disposal, should omit to visit, if only for a few weeks. Its past history and its present fame make it one of the first medical centres of Europe, and, after all, one need not take courses to derive benefit from a period of study and observation spent at any centre.

